# Expansion of Germline Variants in Primary Hyperparathyroidism: Fumarate Hydratase Deficiency as a Cause of Parathyroid Adenomas

**DOI:** 10.1007/s12022-026-09928-w

**Published:** 2026-07-22

**Authors:** Hussam Alkaissi, Elias Chuki, Yi Liu, James Welch, Lynn Bliss, Niharika Shah, Sunita K. Agarwal, William F. Simonds, Martha Quezado, Sanaz Sakiani, Thorkell Andresson, Karel Pacak, Naris Nilubol, Lee S. Weinstein, Christopher A. Febres-Aldana, Smita Jha

**Affiliations:** 1 National Institute of Diabetes and Digestive and Kidney Diseases, National Institutes of Health, Bethesda, MD 20892 USA; 2 Metabolic Diseases Branch, National Institute of Diabetes and Digestive and Kidney Diseases, National Institutes of Health, Building 10, Room 9C432A, 10 Center Drive, Bethesda, MD 20892 USA; 3 Genetics Branch, Center for Cancer Research, National Cancer Institute, National Institutes of Health, Bethesda, MD 20892 USA; 4 Laboratory of Pathology, Center for Cancer Research, National Cancer Institute, Bethesda, MD 20892 USA; 5 Cancer Research Technology Program, Frederick National Laboratory for Cancer Research, National Institutes of Health, Frederick, MD 21701 USA; 6 Section on Medical Neuroendocrinology, Eunice Kennedy Shriver National Institute of Child Health and Human Development, Bethesda, MD 20892 USA; 7 Endocrine Surgery Section, Surgical Oncology Program, National Cancer Institute, Bethesda, MD 20892 USA; 8 Center of Adrenal Endocrine Tumors, AKESO, Prague 5, Czech Republic; 9 Faculty of Medicine, Palacký University, Olomouc, Czech Republic; 10 5th Department of Medicine, Faculty of Medicine, Commenius University, Bratislava, Slovakia

**Keywords:** Heritable primary hyperparathyroidism, Pheochromocytoma, Oncometabolite, Fumarate hydratase, Renal cell cancer, Succinate dehydrogenase

## Abstract

**Abstract:**

A substantial fraction (60–85%) of hereditary primary hyperparathyroidism (hPHPT) lacks an identifiable genetic etiology. We describe fumarate hydratase (*FH*) mutations as a potential cause of hPHPT, expanding the phenotypic spectrum of FH deficiency tumor predisposition syndromes. In an index patient who presented with asymptomatic hypercalcemia and a chief-to-transitional cell-dominant parathyroid adenoma, whole-exome sequencing revealed two unique heterozygous *FH* variants (germline p.Gln376fs*2; somatic p.Pro503_Lys504dup). Functional inactivation of FH was supported by diffuse nuclear and cytoplasmic 2-succinocysteine immunoreactivity and elevated fumarate/malate ratio in tumor tissue. This individual did not show classic HLRCC manifestations. Preserved FH protein expression suggested residual enzymatic activity, which may account for an attenuated phenotype. To assess broader relevance, no additional patients with *bona fide* FH-deficient parathyroid adenoma were identified among 130 individuals with suspected hPHPT of unknown etiology evaluated at our institute. In a complementary cohort of 11 patients with pheochromocytoma/paraganglioma syndrome harboring pathogenic germline heterozygous *FH* variants, one female (*FH* p.Thr234Ala) presented with multi-gland disease requiring parathyroidectomy at age 40 years, features suspicious for hPHPT. These findings support fumarate hydratase deficiency as a plausible etiology for a subset of parathyroid adenomatous disease. Thus, consideration of parathyroid function surveillance in patients with fumarate hydratase deficiency tumor predisposition syndromes may be warranted.

**Clinical Trial Number:**

NCT04969926.

**Supplementary Information:**

The online version contains supplementary material available at 10.1007/s12022-026-09928-w.

## Introduction

Primary hyperparathyroidism (PHPT) is the leading cause of outpatient hypercalcemia and the third most common endocrinopathy with an estimated age-adjusted prevalence rate of 233 per 100,000 in women and 85 per 100,000 in men [1]. Underrecognized PHPT can lead to significant morbidities, including fractures, kidney stones, and chronic kidney disease [2]. Early detection is therefore clinically valuable as parathyroidectomy is the only definitive therapy [3]. Solving the genetic etiology amplifies this benefit because approximately 10–15% of PHPT is heritable (hPHPT) [2]. Germline diagnosis can prevent inappropriate surgery in calcium-sensing disorders.

(for example, in Familial Hypocalciuric Hypercalcemia), guide the extent and timing of surgery in multigland syndromes (e.g. Multiple Endocrine Neoplasia Type 1) and enable cascade testing in family members plus surveillance for extra-parathyroid manifestations. At the molecular level, germline and somatic drivers of PHPT largely converge on two pathogenic axes: (i) dysregulated parathyroid cell growth/neoplasia (often associated with earlier onset, multigland disease, and/or more severe phenotypes), and (ii) altered calcium sensing that shifts the calcium–PTH set point with secondary gland enlargement [2]. Many established hPHPT genes (e.g., *MEN1*,* RET*,* CDKN1B*,* CDC73*,* CASR*,* AP2S1*,* GNA11*,* FLCN*,* GCM2*, and *ZFX)* also contribute to sporadic parathyroid tumorigenesis. Nonetheless, the inherited genetic basis remains unresolved for approximately 60–85% of individuals with suspected hPHPT [2]. This diagnostic gap motivates broader germline- and tumor-based testing approaches, including comprehensive genomic profiling to uncover new predisposition genes and elucidate core regulatory pathways in calcium and phosphate homeostasis.

Fumarate hydratase (FH), also called fumarase, is a crucial enzyme in the Krebs cycle that is expressed as two isoforms via alternative transcription. Translation from p.Met1 produces a mitochondrial form containing a mitochondrial targeting sequence (MTS) that catalyzes fumarate-to-malate conversion in the Krebs cycle. Translation from p.Met44 yields an isoform lacking the N-terminal MTS, resulting in cytosolic localization, where it participates in the DNA damage response and metabolizes fumarate derived from the urea cycle and purine nucleotide cycle [4]. Germline biallelic inactivation of *FH* results in fumaric aciduria, a rare autosomal recessive disorder characterized by systemic inability to convert fumarate to malate, which manifests with severe infantile mitochondrial encephalopathy with hypotonia, seizures, profound developmental impairment, and frequently early childhood mortality [5]. In contrast, heterozygous germline variants present as hereditary leiomyomatosis and renal cell carcinoma (HLRCC), an autosomal-dominant tumor predisposition syndrome characterized by frequent cutaneous leiomyomas, aggressive renal cell carcinoma (RCC), early-onset uterine leiomyomas, and in some patients, pheochromocytoma and/or paraganglioma (PCC/PGL) [6, 7].

PHPT has not been described as part of the tumor predisposition spectrum due to FH deficiency. Here, we present two cases that implicate FH inactivation in the pathogenesis of parathyroid adenomatous disease, expanding the phenotypic spectrum of tumor predisposition due to FH deficiency.

## Materials and Methods

### Study Cohorts

The cases described here were drawn from two cohorts within the National Institutes of Health (Bethesda, Maryland, USA), including the “natural history study of parathyroid disorders” protocol (ClinicalTrials.gov Identifier: NCT04969926) and the “Diagnosis, Pathophysiology, and Molecular Biology of Pheochromocytoma and Paraganglioma” protocol (ClinicalTrials.gov Identifier: NCT00004847). For the present analysis, we included all participants with PCC/PGL in whom an *FH* variant was detected by germline genetic testing and all patients suspected of hPHPT who underwent targeted germline testing for *FH*, as part of efforts to determine the molecular drivers of PHPT. All participants provided written informed consent under the NIH Institutional Review Board (IRB)-approved protocol.

### DNA Sequencing

Whole-exome sequencing (WES) and targeted DNA sequencing with the TruSight Oncology 500 (TSO500) assay were performed through the NCI-COMPASS program. Genomic DNA extracted from macrodissected formalin-fixed paraffin-embedded (FFPE) tumor tissue and matched peripheral blood (germline normal for WES) was sequenced on Illumina NextSeq 550Dx or NovaSeq 6000 instruments (Illumina). Read alignment and variant calling were performed using the Center for Cancer Research Collaborative Bioinformatics Resource (CCBR) Pipeliner workflow (https://github.com/CCBR/Pipeliner*)*, enabling detection of single-nucleotide variants (SNVs), small insertions/deletions (indels), tumor mutational burden (TMB), copy number variants (CNVs), and microsatellite instability (MSI). Copy-number profiles from exome data were inferred using CNVkit v0.8.5 and PureCN v1.8.1 with default parameters.

### Variant Analysis

Coding (exonic) variants were retained for interpretation if they met minimum sequencing quality thresholds (depth of coverage ≥ 20× and variant-supporting read count ≥ 6). Variants were annotated and curated using QIAGEN Clinical Insight Interpret (QIAGEN) and classified according to professional society standards for germline and somatic variant interpretation (ACMG/AMP for germline [8]; AMP/ASCO/CAP for somatic [9]). For germline reporting, a predefined set of 154 cancer-relevant genes was used based on clinical actionability; only pathogenic, likely pathogenic, and variants of uncertain significance (VUS) were returned. For somatic reporting, candidate genes were compiled from the Catalogue of Somatic Mutations in Cancer (COSMIC) and supplemented by focused literature review to prioritize tumor- and disease-relevant oncogenic drivers. All reportable variants were manually reviewed in the Integrative Genomics Viewer (IGV).

### Sanger Sequencing

Parathyroid tumor DNA and blood DNA of patient DK-2005, and a control DNA sample were analyzed by standard PCR and Sanger sequencing to detect LOH for the *FH* variant c.1431_1433dup (p.Lys477dup). The primers used for PCR were hFH-ex10-F attaaccctcactaaagggaTCACTGCTAACCCATATGTCG and hFH-ex10-R aatacgactcactatagggCTTTGGACCCAGCATGTCCTT, with T3 and T7 sequences (shown in lower case), respectively. The 220 bp PCR product was purified and sequenced with the T3 and T7 primer.

### Histology

All parathyroid adenomas in this study were assessed for their predominant cell type—chief cells, oncocytic/oxyphil cells, transitional cells, clear cells, or mixed populations. Architectural growth patterns were also recorded, including solid, follicular, acinar, nested, corded, rosette-like, and papillary configurations. These histomorphologic findings were correlated with the corresponding 2SC labeling patterns.

### Immunohistochemistry

Expression of FH in tumoral cells was evaluated by immunohistochemistry (IHC). Protein succination was evaluated using S-(2-succino)cysteine (2SC), a sensitive surrogate marker of impaired FH enzyme function [10]. IHC was performed on formalin-fixed paraffin-embedded (FFPE) tissue sections from parathyroid tumors using anti-FH (mouse monoclonal, clone J-13, Santa Cruz, Cat# sc-100743, 1:100, RRID: AB_2104094), and anti-2SC (rabbit polyclonal, Discovery Antibodies/Cambridge Research Biochemicals, Cat #crb2005017, 1:2000, RRID: AB_2892588). Antigen retrieval was performed with CC1 for 32 min, followed by 32 min of primary antibody incubation. Staining was carried out on an automated platform using OptiView detection chemistry.

### Immunohistochemisty Scoring

FH and 2SC immunoreactivity were interpreted as previously described [11, 12]. Briefly, for FH, any granular cytoplasmic staining in tumor cells was interpreted as retained expression, whereas complete absence of immunoreactivity was interpreted as loss, with non-neoplastic elements serving as internal positive controls. For 2SC, a reference cohort of 10 parathyroid adenomas, including a subset identified in the setting of Multiple Endocrine Neoplasia Type 1 (MEN1), was used to establish the expected range of 2SC labeling in parathyroid tissues. Adjacent normal parathyroid gland was also evaluated if present. Given that 2SC positivity was consistently observed in the cytoplasm (but never in the nucleus) of oncocytes in both normal and tumoral parathyroid tissues (see Results), we required diffuse cytoplasmic and nuclear 2SC labeling in tumor cells to support aberrant succination consistent with FH deficiency.

### Targeted Metabolome Analysis of FH-deficient Tumors

The concentration of 33 metabolites (see supplemental Table 1) in the central carbon metabolism pathway covering glycolysis, pentose phosphate, TCA and oxidative phosphorylation was measured using targeted multiple reaction monitoring (MRM) assay. Tumor samples were homogenized in 400 µL chilled 80% methanol-water solution using Bead Rupter 12 (Omni International). Supernatant was collected and centrifuged at 14,000 g for 10 min. The metabolite solution was dried in SpeedVac^®^ vacuum concentrator (ThermoFisher Scientific, Waltham, MA) and stored at −20 °C. The remaining pellet was solubilized in Easypep lysis buffer containing DNase (Thermo), and protein estimation performed by bicinchoninic acid (BCA) assay.

All reference target compounds (TGP), for the reversed-phase ion-pairing LC-MS^2^ assay for glycolysis, pentose phosphate, tricarboxylic acid cycle, electron transfer and fatty acid were purchased from Sigma-Aldrich (St. Louise, MO). The stable isotope labeled internal standards (SI-TGP) were ^13^C_3_-lactate, ^13^C_3_-citrate, ^13^C_4_-α-ketoglutaric acid, ^13^C_4_-succinic acid, ^13^C_4_-fumaric acid, ^13^C_4_-malic acid, and ^13^C_5_−2-hydroxyglutaric acid obtained from Cambridge Isotope Laboratory (Andover, MA) as well as ^13^C_6_-glucose-6-phosphate and ^13^C_6_-fructose-1,6-diphosphate purchased from Medical Isotopes, Inc. (Pelham, NH). All TGP and SI-TGP analytical standards have reported chemical and isotopic purity ≥ 98%. OmniSolv^®^ LC-MS grade acetonitrile and methanol were obtained from EMD Millipore (Billerica, MA). Tributylammonium acetate (TBAA) was purchased from TCI America (Portland, OR). LC-MS grade acetic acid and formic acid were obtained from Fisher Scientific (Hampton, NH). All chemicals and solvents used in this study were HPLC or reagent grade unless otherwise noted. Before LC-MS run, samples were reconstituted in 60 µL 3% (v/v) methanol in water, supplemented with 10 µM SI-CCM methanol solution. Ten µL sample was injected for reversed-phase ion-pairing LC-MS2 analysis performed on Thermo TSQ™ Quantiva triple quadrupole mass spectrometer (Thermo Scientific, San Jose, CA) coupled with a NexeraXR LC system (Shimadzu Scientific Instruments, Columbia, MD). Both the HPLC and mass spectrometer were controlled by Xcalibur™ software (Thermo Scientific). The liquid chromatography was carried out on a 100-mm long x 2.1-mm i.d. Synergi Hydro-RP C18 column with 2.5 μm particles and 100 Å pore size (Phenomenex, Torrance, CA) kept at 40 °C. The mobile phase, operating at a flow rate of 200 µL/min, consisted of 10 mM DBAA in water as solvent A and methanol as solvent B. For the analysis of TGP and SI-TGP, a linear gradient stayed at B/A solvent ratio of 3:97 for 3 min, followed by B/A solvent ratio of 80:20 for 14 min. After washing with 98% B for 3 min, the column was re-equilibrated with a mobile phase composition B/A of 3:97 for 10 min prior to the next injection. The general MS conditions were as follows: source: ESI, ion polarity: negative, spray voltage: 2500 V, sheath and auxiliary gas: nitrogen, sheath gas pressure: 40 arbitrary units, auxiliary gas pressure: 5 arbitrary units, ion transfer capillary temperature: 350 °C, scan type: selected reaction monitoring (SRM), collision gas: argon, collision gas pressure: 2 mTorr. The isobaric isomers in this study including CIT/ISOCIT, G6P/F6P, DHAP/GAP, and R5P/XYLU5P, were separated by the LC before tandem mass spectrometry. Quantitation of all metabolites was carried out using Xcalibur™ Quan Browser (Thermo Scientific). Calibration curves for each TGP were constructed by plotting TGP/SI-TGP peak area ratios obtained from the calibration standards curve and fitting the data using linear regression with 1/*X* weighting. The TGP concentrations in samples were then interpolated using the linear function obtained from the calibration curve and normalized against the protein concentration obtained from the pellet after metabolite extraction. Each sample was only analyzed once by single LC/MS run so no technical measurements were performed. Any replication of samples was obtained by splitting tumors during procurement followed by individual processing and analysis. An elevated fumarate to malate ratio (> 1.5) was defined as functional evidence of FH deficiency. Parathyroid tumors from patients with Multiple Endocrine Neoplasia Type 1 (DK-546, DK-2118 and DK-2267) caused by a germline heterozygous inactivating variant in *MEN1* were selected as negative controls as pathogenic variants in *MEN1* explain ~ 40% of “sporadic” parathyroid adenomas and menin loss is an established driver of tumorigenesis in parathyroid tissue [13, 14]. FH-deficient PCC from a patient with pathogenic germline heterozygous *FH* variant served as positive control (run in duplicate). A second run of LC/MS was performed using the same liquid chromatography – mass spectrometer system and methodology as above to confirm the findings from the parathyroid adenoma from DK-2231 and include the parathyroid tumor from DK-2005. MEN1-related parathyroid adenomas (DK-0887, DK-0943, and DK-2047) served as control. Residual FH-deficient PCC from the same patient as in first run served as positive control (run in duplicates).

## Results

### Index Case with Germline *FH* Variant and Primary Hyperparathyroidism

A 38-year-old black female (DK-2231) was noted to have asymptomatic hypercalcemia due to PHPT on routine testing with serum albumin-corrected calcium of 10.4 (ref: 8.4–10.2) mg/dL, serum PTH at 118 (ref: 15–65) pg/mL with replete vitamin D-25-hydroxy (total) levels of 31 (ref: >30) ng/mL and adequate eGFR of 80 ml/min/1.73 m^2^. Her personal history was negative for fractures or kidney stones. Dual-energy X-ray absorptiometry (DXA, Hologic) scan revealed low bone mass with lowest Z-score at the lumbar spine (L1-L4) of −3.1. Fractional excretion of calcium was 1.3%. Her family history was notable for two sisters having undergone hysterectomy for uterine “fibroids” at ages 36 and 45 respectively, but unrevealing for any history of kidney stones, calcium-related derangements, renal cancer, PCC/PGL, or skin tumors. Given her age and low bone mass, parathyroidectomy was recommended. Localization studies including neck ultrasound, ^99m^Tc sestamibi (MIBI) parathyroid scan, and 4-dimensional CT neck (4D-CT) revealed a single heterogeneous, hypoechoic, sestamibi-avid lesion with high vascularity posterior to the left thyroid gland. Targeted hyperparathyroid panel-based germline testing was negative for any variants in *MEN1*,* RET*,* CDC73*,* CDKN1B*,* TRPV6*,* GNA11*,* CASR*, and *AP2S1* (Invitae). The patient underwent superior parathyroidectomy attaining surgical cure with an adequate drop in intra-operative PTH. The resected tissue consisted of a large parathyroid adenoma, measuring 5.2 × 2.4 × 1.8 cm with a weight of 6.44 g with a surrounding rim of normal parathyroid tissue [15].

### Detection and Curation of Germline *FH* Variants

The patient’s (DK-2231) relatively young age at presentation was suspicious for heritable forms of PHPT and hence, matched tumor-normal whole exome sequencing was performed. Germline peripheral blood sample molecular testing revealed a likely pathogenic heterozygous variant in *FH* (NM_000143.4) c.1127_1128delAG (p.Gln376fs*2). No additional pathogenic variants or variants of uncertain significance (VUS) were identified. This frameshift variant creates a premature translational stop signal; the cDNA level change is two nucleotide deletion in exon 8 resulting in an absent or disrupted protein product. This variant is extremely rare in the general population, with an allele frequency of 1/1,614,106 in the Genome Aggregation Database (gnomAD v4.1.0). The alteration is predicted to result in loss of function through premature protein truncation or nonsense-mediated mRNA decay. Loss-of-function variants in *FH* are a well-established mechanism of pathogenicity, and this variant is classified as pathogenic in ClinVar (Variation ID: 2573255; accessed January 16, 2026). The variant has been reported in at least one patient with HLRCC [16]. The presence of pathogenic germline heterozygous variants in *FH* is consistent with a diagnosis of hereditary HLRCC. The patient denied any personal history of HLRCC-related manifestations. A comprehensive skin exam revealed no cutaneous leiomyomas. No renal or uterine mass was noted on magnetic resonance imaging (MRI) of the abdomen and pelvis. Rare association of pheochromocytoma has been described in patients with HLRCC [17, 18]. Patient’s biochemical (plasma fractionated metanephrines and catecholamines) and imaging (CT adrenals) evaluation was negative for PCC/PGL.

### Somatic *FH* Alterations in Parathyroid Tumors

Whole exome sequencing of parathyroid tumor sample of patient DK-2231 revealed a VUS in *FH* c.1506_1511dupACCTAA (p.Pro503_Lys504dup), with an allele frequency of 36% (estimated tumor content 70%) in addition to the germline variant (allele frequency in the tumor was 48%). No additional pathogenic small nucleotide variants or reportable fusions or rearrangements were identified in the tumor including in any of genes known to cause parathyroid tumors such as *CDC73*,* MEN1*,* RET*,* CDKN1B*,* CASR*,* AP2S1*,* GNA11*,* FLCN*,* GCM2* and *ZFX*. Additional somatic VUS identified in the tumor included *ACIN1* (c.37 A > C, p.Thr13Pro; allele frequency 12%), *FAM188A* (c.3544 C > A, p.Pro1182Thr; allele frequency 13%), *NRXN1* (c.2569c > T, p.Arg857Cys; allele frequency 21%) and *OR11H2* (c.670 C > A, p.Leu224Ile; allele frequency 20%). None of these genes are known to result in tumor predisposition. The somatic *FH* variant p.Pro503_Lys504dup is an in-frame duplication resulting from the tandem duplication of two amino acids near the C-terminus of the protein. It is not reported in cBioPortal or in COSMIC and is classified as a VUS in ClinVar (Variation ID: 405910). In this tumor, identification of a germline pathogenic *FH* variant together with a somatic *FH* variant of uncertain significance raised the possibility of a *FH*-deficient parathyroid neoplasm.

### Prevalence of Germline *FH* Variants in Patients with Primary Hyperparathyroidism

Based on these findings, we reviewed available reports from panel-based (including *FH*) germline genetic testing (Invitae custom panel) in 130 patients with suspected heritable forms of hyperparathyroidism (positive family history, multi-gland disease, age of onset ≤ 45 years) who were evaluated at our center and had tested negative for variants in genes known to be associated with PHPT (Invitae hereditary hyperparathyroidism panel). This resulted in the identification of one additional patient (DK-2005) with germline heterozygous likely pathogenic variant in *FH* c.1431_1433dup (p.Lys477dup) in the fumarase domain of the gene. The variant has conflicting classifications of pathogenicity in ClinVar (Variation ID: 42095) and is observed in general population at a relatively elevated minor allele frequency (MAF) of approximately 0.1% (gnomAD v4.1). This variant has been identified in fumarase deficiency in the compound heterozygous state [19–22]. Functional studies across several independent reports demonstrated reduced enzyme activity [20, 21, 23, 24], however, only a single study has documented impaired enzymatic function in the absence of a second pathogenic *FH* variant [24]. Multiple large studies suggest that the variant is not associated with HLRCC [25, 26]. Collectively, these findings indicate that while the variant may contribute to reduced fumarase activity, its role as a high-penetrance cancer-predisposition allele remains unproven. Next generation sequencing using targeted TruSight Oncology 500 gene panel did not reveal a second hit in *FH* in the parathyroid tumor from DK-2005. Sanger sequencing of patient’s parathyroid tumor DNA was also negative for loss of heterozygosity at the *FH* locus (Supplemental Fig. 1).

### Immunohistochemical Evaluation of Fumarate Hydratase Deficiency in Parathyroid Tissues

**Fig. 1:**
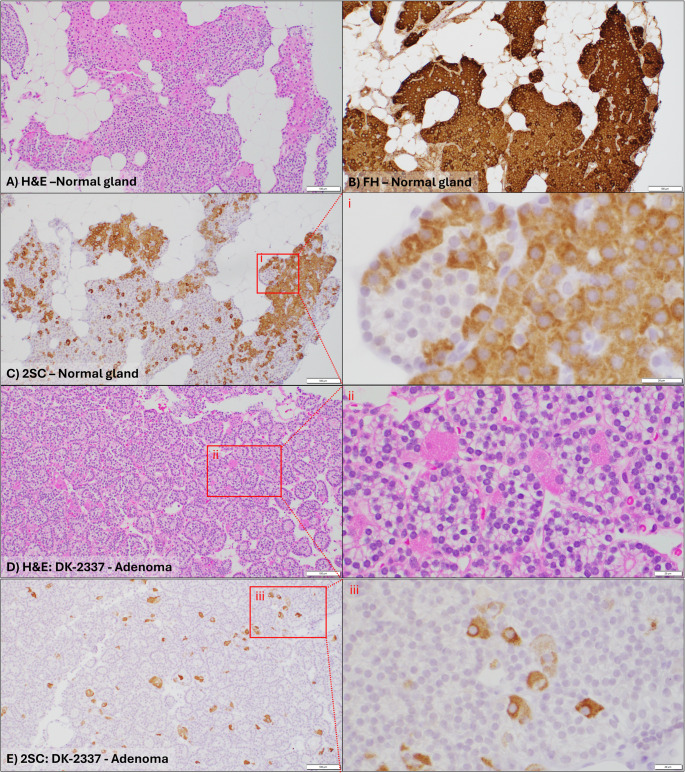
FH and 2SC immunohistochemistry in FH-intact parathyroid tissues. (**A**) Normal parathyroid gland with retained FH expression (**B**) and focal 2SC immunoreactivity in oncocytic cells (**C**; inset i: cytoplasmic labeling confined to oncocytes [right], with adjacent chief cells negative [left]). (**D**) Parathyroid adenoma from a patient with sporadic primary hyperparathyroidism (DK-2337) showing a chief cell–predominant neoplasm with scattered oncocytes (inset ii) and corresponding 2SC staining (**E**; inset iii: weak, cytoplasmic-only labeling restricted to oncocytic cells)

**Fig. 2:**
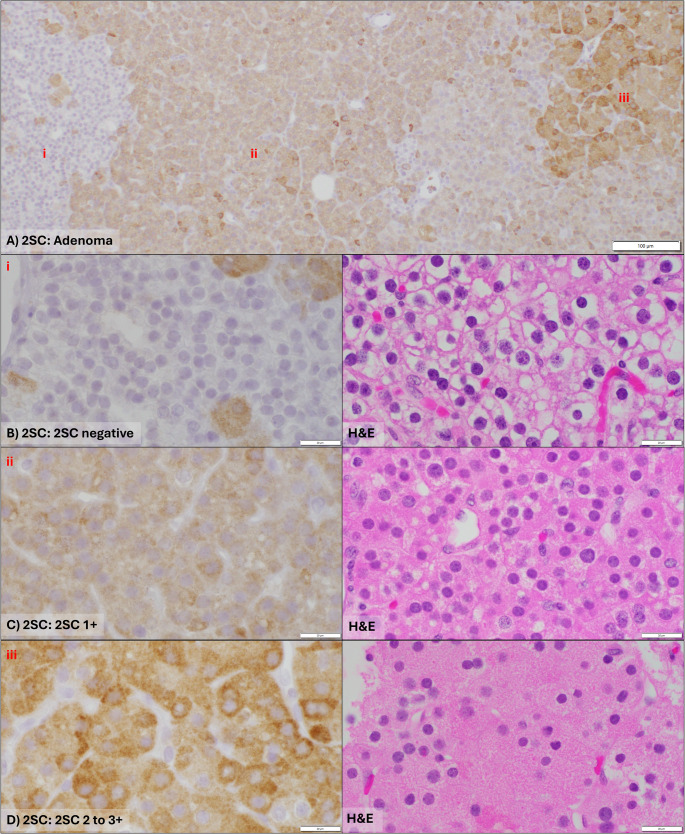
Variable 2SC labeling in an oncocytic parathyroid adenoma (DK-2327). (**A**) Low-power view demonstrating intratumoral heterogeneity in 2SC staining, with negative (i), weakly positive (ii), and strongly positive (iii) regions; corresponding high-power views are shown in (B)–(D), respectively. (**B**) 2SC-negative component, composed of chief cells. (**C**) Weak (1+), cytoplasmic-only 2SC labeling in transitional oncocytes

2SC (2-succinyl cysteine) IHC is a highly sensitive and specific biomarker for detecting FH-deficient tumors reflecting underlying metabolic derangements caused by FH loss. FH deficiency results in accumulation of fumarate, which covalently modifies cysteine residues in proteins to form 2SC, a post-translational modification which is not typically seen in normal tissues and FH-replete tumors [10, 27–29]. However, aberrant succination can also occur in “pseudohypoxic environments” and mitochondrial dysfunction that drives fumarate accumulation and subsequent protein succination, even with intact FH [30–32]. To establish a range of 2SC labeling expected for FH- intact parathyroid tissues, ten parathyroid adenomas and adjacent parathyroid gland were evaluated for 2SC and FH IHC. The clinico-pathologic characteristics and IHC results are summarized in Table 1 2SC immunoreactivity was observed in all adenomas, irrespective of familial/MEN1 (*n* = 5) or sporadic (*n* = 5) status, and in adjacent non-neoplastic parathyroid tissue when present (*n* = 7). In all cases, 2SC labeling paralleled oncocytic differentiation: oncocytic/oxyphil cells were uniformly positive, and the overall proportion of 2SC-positive cells reflected the fraction of oncocytes within the lesion. In contrast, chief and clear cells were consistently negative. Across this reference cohort, oncocytic staining was consistently weak (1+) and confined to the cytoplasm (Fig. 1A and E). Notably, one control case (DK-2327) showed a spectrum of oncocytic change, and 2SC intensity increased with the degree of cytoplasmic expansion; however, labeling remained restricted to the cytoplasmic compartment (Figs. 2A–D).

**Table 1:** Fumarate Hydratase and 2-succino cysteine Immunohistochemistry in Parathyroid Tissue

Case	Presentation	Parathyroid Adenoma							Normal Parathyroid	
		Growth Pattern	Dominant cell type	Oncocytes (%)	FH IHC status	2SC			2SC	
						%	Intensity	Pattern	%	Pattern
DK-2308	Sporadic	Solid	Chief	1-5	Retained	1	1+	Cytoplasmic	10	Cytoplasmic
DK-1208	MEN1	Solid	Mixed	50	Retained	50	1+	Cytoplasmic	20	Cytoplasmic
DK-1247	MEN1	Acinar	Mixed	40	Retained	40	1+	Cytoplasmic	5	Cytoplasmic
DK-1574	Sporadic	Acinar	Mixed	40	Retained	40	1+	Cytoplasmic	NA	NA
DK-1577	MEN1	Nested	Chief	1-5	Retained	2	1+	Cytoplasmic	10	Cytoplasmic
DK-2327	Sporadic	Solid	Oncocytic	95	Retained	95	1+ to 3+	Cytoplasmic	30	Cytoplasmic
DK-2337	Sporadic	Nested	Chief	1-5	Retained	2	1+	Cytoplasmic	40	Cytoplasmic
DK-2339	MEN1	Solid	Chief	1-5	Retained	2	1+	Cytoplasmic	NA	NA
DK-2331	Sporadic	Solid	Chief	30	Retained	30	1+	Cytoplasmic	10	Cytoplasmic
DK-2319	Heritable	Solid	Chief	1-5	Retained	2	1+	Cytoplasmic	NA	NA
DK-2231	Pathogenic FH variant	Acinar/Nested	Chief	1-5	Retained	100	3+	Nuclear and Cytoplasmic	10	Cytoplasmic
DK-2005	FH variant – uncertain pathogenicity for HLRCC	Nested	Chief/Clear	1-5	Retained	30	1+	Cytoplasmic	10	Cytoplasmic

*FH* fumarate hydratase, *2SC* S-(2-succino) cysteine. Intensity: 1+ (weak), 2+(moderate), 3+(strong)

**Fig. 3:**
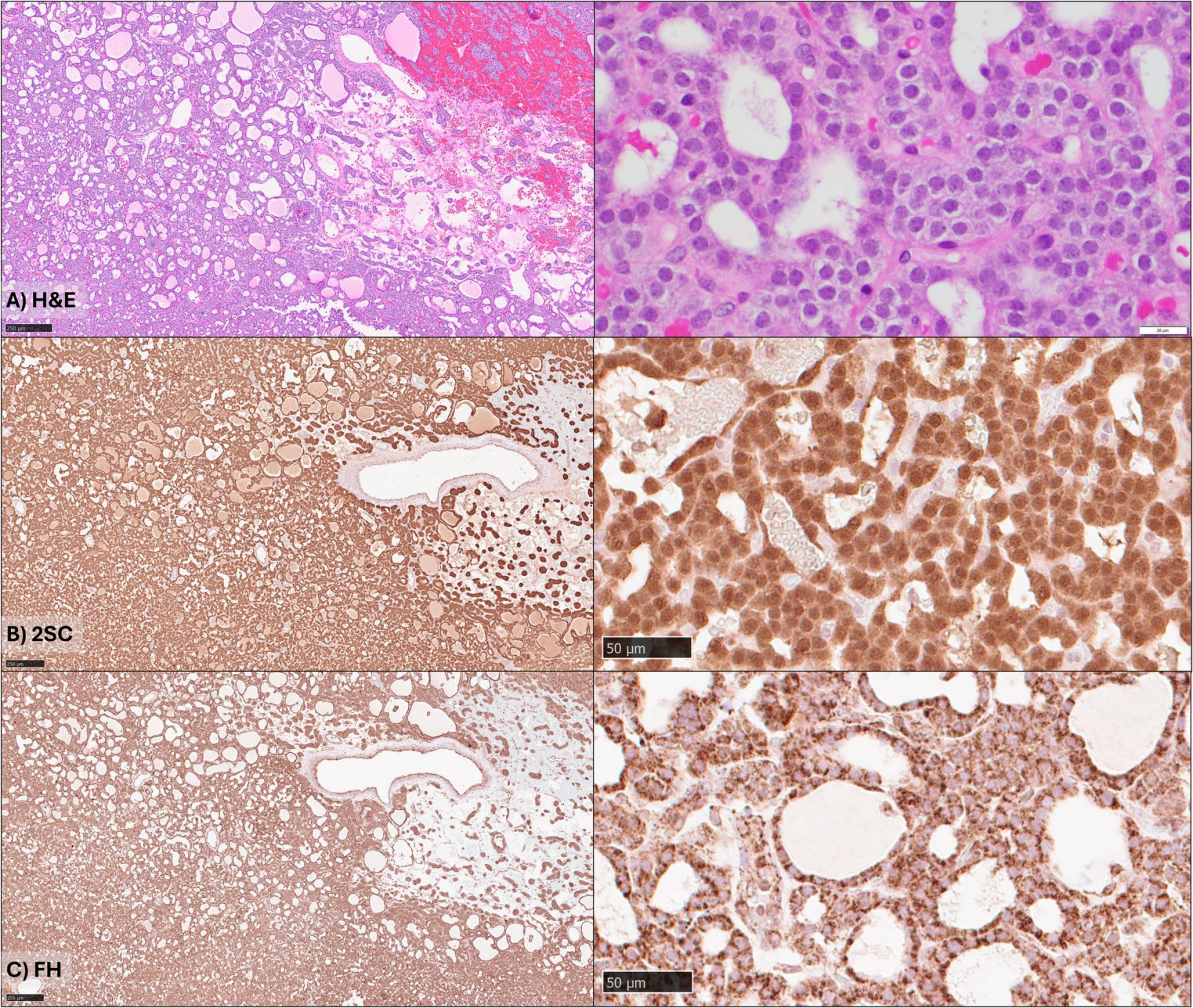
2SC labeling supporting FH-deficiency in a parathyroid adenoma (DK-2231). (**A**) Low-power view (left) of the H&E-stained section of a parathyroid adenoma in acinar and nested pattern with focal microcystic change. High-power view (right) showing predominantly chief-to-transitional cells with slightly granular cytoplasm. (**B**) Low-power (left) and high-power (right) views of 2SC showing diffuse, strong labeling of cytoplasm and nuclei. (**C**) Low-power (left) and high-power (right) views of FH showing retained expression. (**D**) Stronger cytoplasmic 2SC labeling in *bona fide* oncocytes

**Fig. 4:**
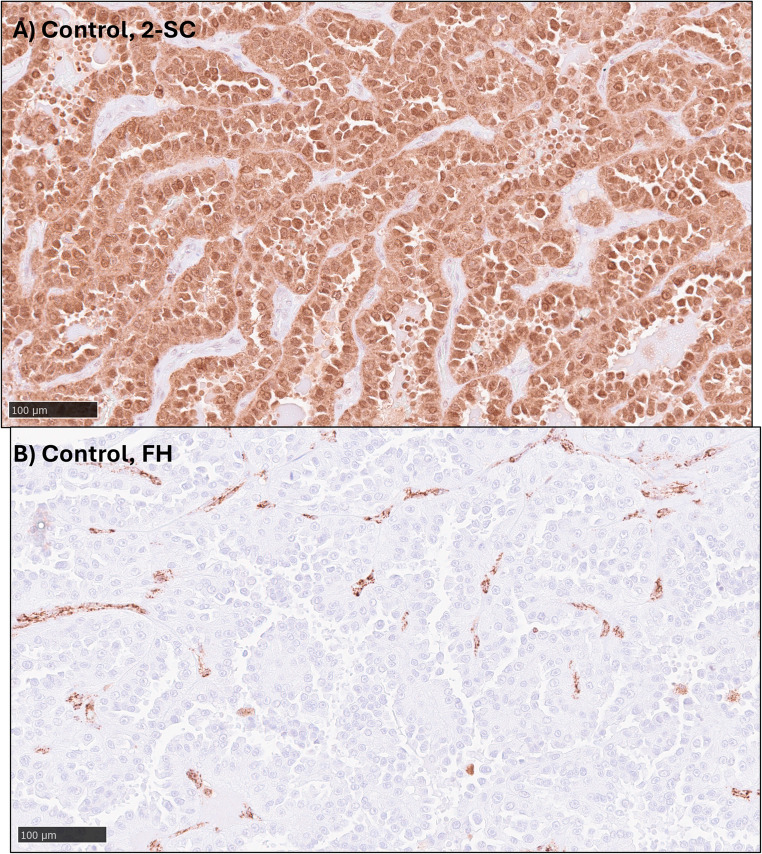
Immunohistochemistry for 2SC and FH in a FH-deficient renal cancer specimen.**A**) Strong, diffuse nuclear and cytoplasmic 2-succinocysteine labeling in a fumarate hydratase-deficient renal cancer specimen from a patient with Hereditary Leiomyomatosis and Renal Cell Cancer. **B**) Immunohistochemistry for fumarate hydratase was negative showing loss of expression with positive internal control (endovascular cells)

**Fig. 5:**
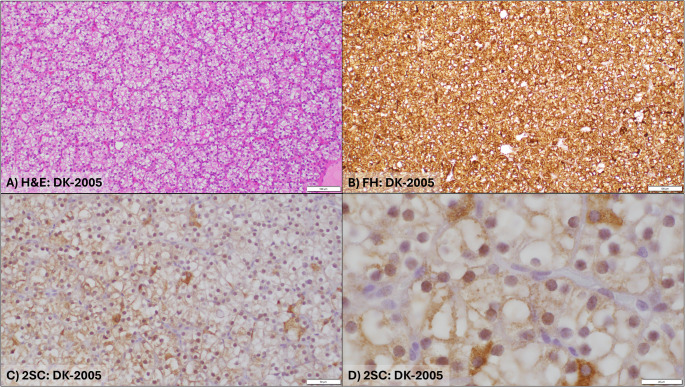
Functional characterization of FH in parathyroid adenoma in patient with germline *FH* c.1431_1433dup (p.Lys477dup).**A**) Parathyroid adenoma from patient DK-2005 with a nested architecture composed of chief and clear cells, with no atypical features. **B**) Immunohistochemistry revealed retained expression for fumarate hydratase and 2-succinocysteine showed only limited cytoplasmic staining (**C**) without definitive nuclear labeling to support FH deficiency (**D**)

The resected parathyroid tumor from patient DK-2231 was an adenoma with acinar and nested growth patterns, composed predominantly of chief cells, a subset of which exhibited slightly granular cytoplasm consistent with transitional oncocytic change (Fig. 3A). No atypical features, such as mitotic figures, necrosis, invasive growth or desmoplasia were identified. FH expression was retained; however, 2SC demonstrated strong, diffuse nuclear and cytoplasmic labeling in tumor cells, consistent with aberrant succination characteristic of other FH-deficient tumors (Fig. 3B and C; Fig. 4). This case supports requiring nuclear staining (in addition to cytoplasmic labeling) in parathyroid tumors to interpret 2SC as aberrant and indicative of FH deficiency. The tumor excised from patient DK-2005 was an adenoma with a nested architecture composed of chief and clear cells, with no atypical features. FH expression was retained, and 2SC showed only limited cytoplasmic staining without definitive nuclear labeling (Fig. 5).

### Metabolomic Confirmation of Fumarate Hydratase Deficiency

**Fig. 6:**
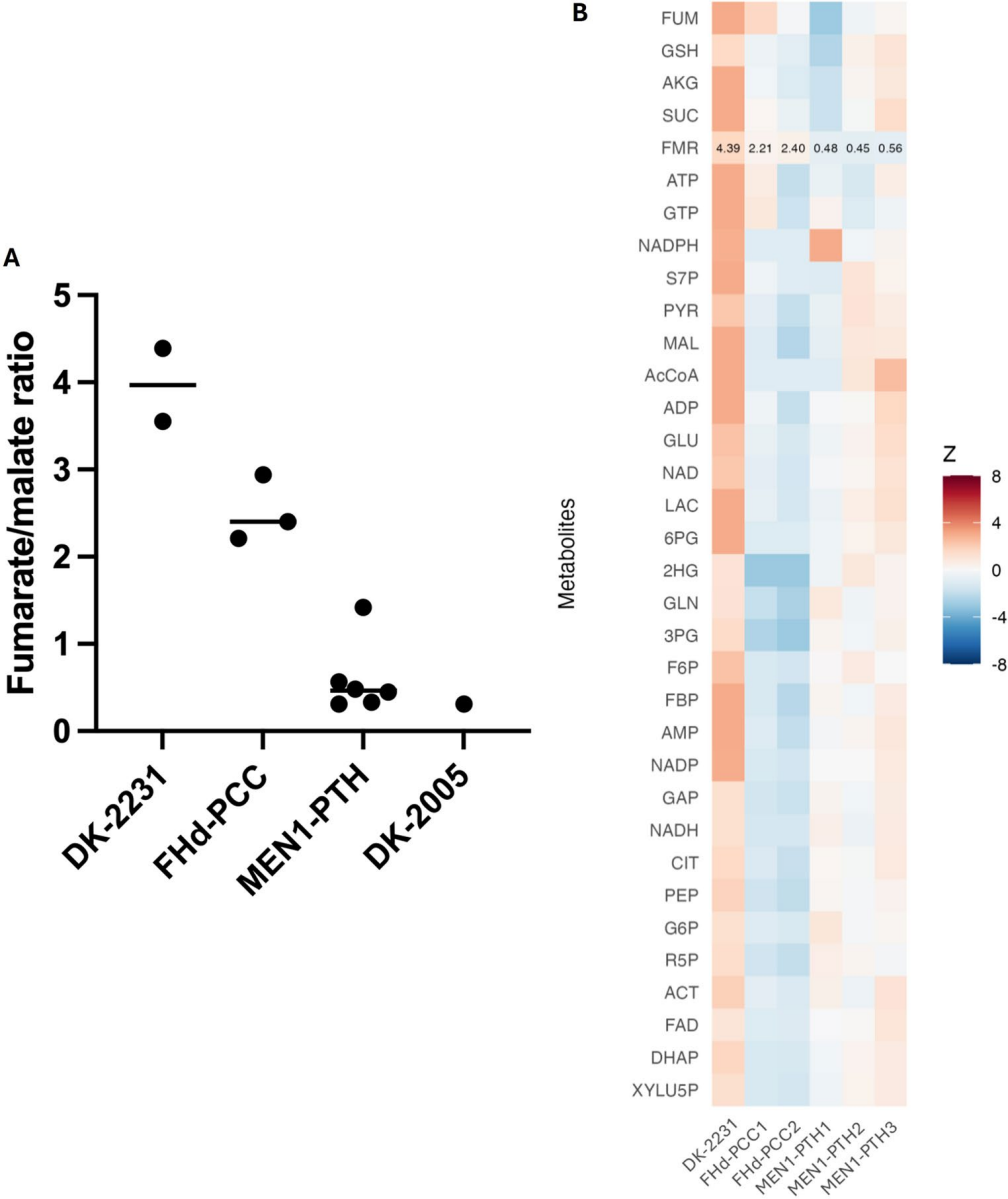
Metabolomic analysis of parathyroid tumors.**A**) Bar graph showing the highest concentration of protein-adjusted fumarate/malate ratio in the FH-deficient parathyroid tumor (DK-2231), relative to FH-deficient paraganglioma (FHd-PCC positive control from single patient) and MEN1-related parathyroid adenoma (MEN1-PTH; negative control, six samples). Data from both batches of samples combined. **B**) Heat map of the targeted metabolome analysis measuring concentration of metabolites of glycolysis, tricarboxylic acid cycle, pentose phosphate pathway and oxidative phosphorylation and redox in tumor tissue through mass spectrometry showing the clustering of FH-deficient parathyroid adenoma (DK-2231) with FH-deficient paraganglioma in duplicate (FHd-PCC1 and FHd-PCC2, positive control). The highest protein-adjusted concentration of fumarate was seen in FH-deficient parathyroid adenoma. Three MEN1-related parathyroid adenomas (MEN1-PTH 1, 2 and 3 served as negative control. Data (representative) only shown for the first batch. Raw data from the two batches of the analysis are included in the supplement. The more intense red color denotes the higher amount while blue represents lower amount of the metabolite. *Note*. *FUM* fumarate,*GSH* glutathione, *AKG* 2-oxoglutarate, *SUC* succinate, *FMR* fumarate malate ratio, *ATP* adenosine triphosphate, *GTP* guanosine triphosphate, *NADPH* nicotinamide adenine dinucleotide phosphate (reduced), *S7P* sedoheptulose-7-phosphate, *PYR* pyruvate, *MAL* malate, *AcCoA* acetyl-coenzyme A, *ADP* adenosine diphosphate, *GLU* glutamate, *NAD*: nicotinamide adenine dinucleotide, *LAC* Lactate, *6PG* 6-phosphogluconate, *2HG* 2-hydroxyglutaric acid, *GLN* glutamine, 3PG 3-phosphoglycerate, *F6P* fructose-6-phosphatem, *FBP* fructose-1,6-diphosphate, *AMP* adenosine monophosphate, *NADP* nicotinamide adenine dinucleotide phosphate, *GAP* glyceraldehyde 3-phosphate, *NADH* nicotinamide adenine dinucleotide (reduced), *CIT* citrate, *PEP* phosphoenolpyruvate, *G6P* glucose-6-phosphate, *R5P* ribose-5-phosphate, *ACT* cis-aconitate, *FAD* flavin adenine dinucleotide, *DHAP* dihydroxy-acetone-phosphate, *XYLU5P* xylulose-5-phosphate

We conducted a targeted metabolomic analysis employing liquid chromatography-mass spectrometry (LC-MS/MS) based metabolomics of fresh-frozen tumor specimen from patient, DK-2231 (two separate runs), DK-2005, six patients with MEN1-related parathyroid tumors (negative control), and one patient with FH-deficient pheochromocytoma in the context of a germline *FH* variant (positive control, in replicates). The parathyroid tumor from DK-2231 showed the highest protein-adjusted concentration of fumarate and fumarate/malate ratio (Fig. 6A and B). An enrichment in the glycolysis-related metabolites was observed in the parathyroid tumor and the two replicates of FH-deficient pheochromocytoma. This shift or Warburg effect is a well-recognized phenomenon of FH-deficient tumors to use glycolysis as their source of energy due to TCA disruption from FH loss. To note, depletion of glutathione was not observed in the FH-deficient parathyroid tumor. Overall, patient DK-2005’s parathyroid tumor with germline variant *FH* c.1431_1433dup (p.Lys477dup) did not exhibit aberrant FH/2SC labeling or an elevated fumarate/malate ratio and no second hit to the gene was identified on targeted tumor sequencing or Sanger. Hence, there is no evidence to support FH-deficiency in her parathyroid tumor.

### Prevalence of Primary Hyperparathyroidism in Patients With Germline *FH* Variants

**Fig. 7:**
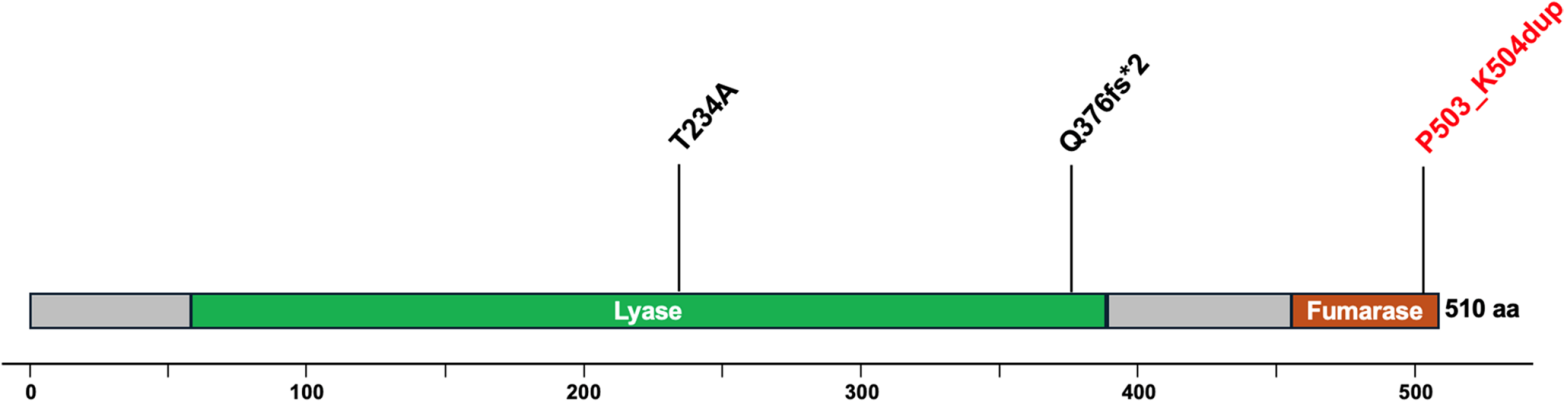
Figure depicts the schema of the FH protein domains and location of the germline and somatic *FH* variants identified in patients with parathyroid adenomas. Pathogenic variants p.Q376fs*2, and p.T234A were identified in the germline of patients DK-2231 and CH-01278 respectively while the variant p.P503_K504dup was identified in the parathyroid adenoma from DK-2231 – labeled as variant of uncertain significance but shown to be pathogenic in this report through immunohistochemistry for 2-succinocysteine (2SC) and demonstration of high concentration of fumarate and fumarate/malate ratio on metabolomic analysis of the tumor. Black: germline variant; Red: somatic variant

Among patients with pheochromocytoma/paraganglioma (PCC/PGL), germline variants in *FH* account for 3.1% of patients [17]. Our centre has an active research program in PCC/PGL which allowed us to identify 11 patients with germline pathogenic variants in *FH*, all of whom had PCC/PGL. We performed a chart review of these patients for past medical history or biochemical profile. Of these, one patient (CH-01278) with germline pathogenic variant *FH* c.700 A > G, p.Thr234Ala was found to have a past medical history of PHPT which occurred several decades prior to her presentation at our institute [17, 18]. This patient, a 60-year-old female had previously undergone a left adrenalectomy at age 39 years for a 7 cm pheochromocytoma, followed by local recurrence and metastasis at age 59 years. In addition, she had a total hysterectomy at age 45 years for uterine leiomyomas. Family history was notable for multiple family members with pheochromocytoma. Targeted germline genetic testing for *MEN1* and *RET* was negative. Subsequent whole-genome sequencing identified a pathogenic *FH* variant (c.700 A > G; p.Thr234Ala). Of note, she underwent three-gland parathyroidectomy for PHPT at age 40 years at an outside institution. At her last follow-up at age 69 years, the patient showed no evidence of recurrent PHPT, and her serum calcium level remained within normal limits at 2.49 mmol/L [ref: 2.15–2.55 mmol/L] without any calcium supplementation. The finding of multi-gland disease and relatively young age of PHPT onset are highly suspicious for hPHPT, likely FH-deficient (Fig. 7). Unfortunately, no tissue was available for additional testing.

## Discussion

In this report, we describe Krebs cycle defects as the cause of parathyroid adenomas in 1/131 (< 1%) patients with suspected hPHPT, 1/11 (9%) patient with germline *FH* variants and PCC/PGL. These findings expand the clinical spectrum of the tumor predisposition syndrome due to FH deficiency to include PHPT and introduce fumarate as the first oncometabolite to result in parathyroid tumors. Thus, our findings have implications for management of patients with tumor predisposition syndrome due to FH deficiency and for patients with hPHPT of unknown cause.

*FH* is located on chromosome 1q43 and its main functional domains include: the N-terminal (residues 1–58) and central (residues 59–389) lyase I, and C-terminal (residues 455–509) fumarase C, with pathogenic variants tending to cluster in the central and C-terminal domain [33, 34]. The active enzyme in humans is a homo-tetramer. Penetrance for RCC is incomplete and variable with a lifetime risk of 10–15% [35–37]. Notably, none of the patients with germline *FH* variant reported in this report have developed renal cancer to date. No genotype phenotype correlation has been reported in the disease, and no specific *FH* variant or domain is reliably predictive of clinical severity, tumor risk, or organ involvement [6]. Identical *FH* variants have been described in families with and without RCC [6]. Missense *FH* variants are noted to confer an increased risk of PCC/PGL, but not necessarily RCC [17, 18]. The lack of development of RCC in patients described above likely reflects its incomplete penetrance, tissue-specific susceptibility or possible phenotype-genotype correlation. FH-deficient RCC is characterized by aggressive clinical behavior, often presenting with larger tumors and higher stage at diagnosis with frequent early metastasis and rapid progression, early onset, and marked morphologic heterogeneity [38, 39]. Approximately 50% of patients with HLRCC die within a median follow-up of 16 months [40]. Similarly, FH-deficient PCC/PGL tend to be more aggressive with a high metastatic potential [18, 41]. In contrast, FH-deficient leiomyomas in the skin and uterus are multiple but benign. We did not observe a distinct clinical pattern with the parathyroid tumor(s) described in this report however, identification of additional patients with parathyroid tumors due to Krebs-cycle defects will help in this assessment. Recent report of PHPT in a 24-year-old male patient with Ollier Disease from *IDH1* mosaicism (p.Arg132Leu) at an allele frequency of 16% diagnosed on sequencing a hand enchondroma sample is noted although no functional studies were performed in the parathyroid tumor [42].

*FH* loss is known to result in tumorigenesis in renal tissue through multi-factorial mechanism. First, the accumulated succinate and fumarate result in wide-spread inhibition of α -ketoglutarate-dependent dioxygenases, such as prolyl hydroxylases (PHD), Ten-Eleven Translocation (TET) and other enzymes. Inhibition of PHDs result in stabilization of hypoxia-inducible factor (HIF-1α and HIF-2α) resulting in activation of transcriptional profile that promotes angiogenesis, anaerobic glycolysis, epithelial-mesenchymal transition, and other oncogenic effects [24, 43]. Accumulation of succinate and fumarate in mouse and human cells has been shown to result in inhibition of dioxygenases such as Ten-Eleven Translocation (TET)-mediated demethylases resulting in epigenetic changes and histone hypermethylation [44, 45]. These epigenetic changes can explain the epithelial-to-mesenchymal transition signature and enhanced migratory and invasive properties seen in FH-deficient RCC [44]. Additionally, fumarate has unique deleterious effects. Given its double bond between C2 and C3, fumarate acts as an electrophile, and can spontaneously react with the thiol group of free cysteines or cysteine residues in glutathione and proteins in a process called succination. Gene expression profiling show HIF-independent fumarate-mediated succination of proteins resulting in their dysregulation as the candidate oncogenic pathway in mice with Fh1-deficient renal cysts [46, 47]. It is worth noting that fumarate and succinate do not completely overlap in their downstream metabolic effect. The presence of double bond in the backbone of fumarate distinguishes it from succinate, and makes fumarate an electrophile (electron acceptor), and reactive, binding to other proteins as a 2-succinocystein. As such, while both succinate and fumarate function as competitive inhibitors of alpha ketoglutarate dependent dioxygenases, fumarate has an additional layer in its pathogenicity in the form of protein succination, impacting several pathways such as Kelch-like-ECH-associated protein 1(KEAP1)-pathway [48]. Fourth, gene expression studies of renal cysts from *Fh1*-deficient mice and HLRCC show an upregulation of glycolytic genes consistent with metabolic reprogramming due to Warburg effect, correlating with and potentially contributing to tumorigenesis [49, 50]. Finally in vitro studies have shown that the cytosolic FH isoenzyme has a key role in DNA damage response and its deficiency results in increasing genomic instability promoting tumorigenesis [51, 52]. Whether these mechanisms are relevant in the parathyroid tissue remains to be evaluated. Lack of in vitro models and parathyroid cell line limit our ability to evaluate this further.

*FH* adds to a growing list of genes which include *CDC73* and *FLCN* that are associated with both renal and parathyroid tumors [53]. Some similarities between chief cells and distal nephron tubular cells (the assumed cell of origin in FH-deficient renal cell cancer [39, 40]) include: (i) both are polarized epithelial cells with apical-basal organization for secretion and absorption respectively (ii) both are highly metabolically active, ion-sensing cells and (iii) both are involved in calcium homeostasis with chief cells secreting PTH and distal nephron tubular cells having a key role in PTH-dependent calcium reabsorption. Furthermore, oxyphil cells of parathyroid gland which can also secrete PTH and predominate with aging and most cells of the kidney including distal nephron tubular cells are enriched in mitochondria, the cellular site of TCA cycle. Similarly, the adrenal medulla and parathyroid glands share some commonalities. Both have significant neural crest contributions to their development. While chromaffin cells are directly derived from neural crest cells, neural crest-derived mesenchymal cells surround the pharyngeal pouch endoderm and parathyroid primordium, invade the parenchyma, and are essential for proper gland development and patterning [54–56]. Additionally, both cell types belong to the amine precursor uptake and decarboxylation cell series sharing distinctive neuroendocrine characteristics such as chromogranin A expression, utilizing calcium-regulated exocytosis for hormone release and storing hormones in dense core secretory granules [57–61]. Developmentally, chromaffin cells, parathyroid cells, and renal cells use GATA3 as a shared lineage-defining transcription factor [62, 63].

Our work demonstrates that FH functions as a critical tumor suppressor in parathyroid tissue. Our report has some limitations. First, the short-read next-generation sequencing platforms used in this study do not permit phasing of variants separated by the distance observed in this case. This limits our ability to establish *bone fide* biallelic events. Resolving the allelic configuration would require long-read sequencing technologies. Nevertheless, functional characterization of the tumor, including demonstration of aberrant 2-succinocysteine (2SC) on IHC and accumulation of the oncometabolite fumarate, reflected by a four-fold elevation in fumarate-to-malate ratio supports FH deficiency. This metabolomic signature was not observed in MEN1-related parathyroid adenomas, supporting a specific association with FH dysfunction rather than a nonspecific feature of parathyroid neoplasia.

Second, although we established an expected range of 2SC labeling in FH-intact parathyroid tissues, our findings suggest that cytoplasmic-only 2SC labeling in oncocytes warrants further investigation. Oncocytic tumors are characterized by mitochondrial dysfunction, and 2SC immunoreactivity may reflect the burden of mitochondrial mutations in oncocytes in both non-neoplastic parathyroid tissue and parathyroid neoplasia [64–66]. In the current study, we therefore defined diffuse 2SC labeling involving both the cytoplasm and nuclei as the most plausible surrogate marker of FH deficiency in parathyroid pathology. We emphasize that FH/2SC staining patterns must be interpreted in a tissue-specific context. For example, FH-deficient renal tumors typically show diffuse, strong nuclear and cytoplasmic 2SC staining, whereas tumors without FH deficiency may exhibit patchy or diffuse cytoplasmic-only staining without nuclear labeling, with the caveat that renal tumors may also be enriched for mitochondrial mutations [27, 29]. Retained FH immunoreactivity in the setting of positive 2SC staining, similar to the pattern observed in DK-2231, has been described in uterine and cutaneous leiomyomas and in renal cell carcinoma, and has been interpreted as indicating a dysfunctional FH protein detectable by IHC [11, 67–69].

Third, we did not assess the prevalence of somatic biallelic FH loss, as only germline profiling was performed for FH variants in 131 patients with suspected hPHPT. Fourth, the observed metabolomic profile (elevated fumarate, increased fumarate-to-malate ratio, and enrichment of glycolytic metabolites) could conceivably represent a more general metabolic feature of parathyroid adenomas [70]. However, the absence of fumarate-to-malate ratio elevation in six independent MEN1-related parathyroid tumors and in the tumor from patient DK-2005 makes this an unlikely proposition. We acknowledge, however, the relatively small number of control parathyroid tumors (*n* = 10) and that the control set was not enriched for oxyphilic-predominant lesions. Oxyphil cells are characterized by abundant mitochondria, with mitochondrial DNA content increasing by ~ 2.5-fold from chief to oxyphil cells [64]. Accordingly, we cannot exclude somatic mitochondrial DNA variants affecting NADH dehydrogenase (complex I) or cytochrome c oxidase (complex IV), which could secondarily promote fumarate accumulation through Krebs cycle inhibition, in the DK-2231 tumor [64, 71]. Nonetheless, most renal oncocytomas, the prototypical oncocytic neoplasms with abundant, dysfunctional mitochondria, do not show 2SC labeling or elevated fumarate levels, suggesting that oncocytic morphology alone is insufficient to account for the metabolomic changes observed in the FH-deficient parathyroid tumor from DK-2231 [67].

In conclusion, Krebs cycle defects and consequent oncometabolite accumulation are associated with metabolic reprogramming and tumorigenesis in parathyroid tissue. Whether or not the patients with germline *FH* variants who develop FH-deficient parathyroid tumors eventually develop RCC remains to be evaluated but at the present time surveillance for HLRCC and cascade screening is recommended in all individuals with a germline *FH* variant [72]. Similarly, monitoring of the parathyroid function in patients with Krebs cycle defects who present with HLRCC or PCC/PGL would be useful in enhancing our understanding of parathyroid tumorigenesis. Approximately 65–80% of patients with heritable (“familial isolated”) hyperparathyroidism remain genetically unexplained. These findings advance our understanding of the molecular mechanisms of the parathyroid gland(s) and its tumorigenesis and opens the possibility of targeting the metabolic and signaling consequences of oncometabolite accumulation in patients for whom parathyroidectomy may not be suitable. Future research to characterize the metabolic signature of parathyroid tumors is warranted.

## Supplementary Information

Below is the link to the electronic supplementary material.

Supplementary Material 1

## Data Availability

Some but not all data are available upon reasonable request to the corresponding author. Data sharing will require evaluation of the request by the local Research Ethics Board and the signature of a data transfer agreement.
