# Supplementary material for: Expansion of Germline Variants in Primary Hyperparathyroidism: Fumarate Hydratase Deficiency as a Cause of Parathyroid Adenomas
Source: Endocr Pathol. 2026 Jul 22;37(1):32. doi: 10.1007/s12022-026-09928-w (PMC13391723; doi:10.1007/s12022-026-09928-w)
Supplement: Supplementary file 1 — Supplementary Material 1 [file 12022_2026_9928_MOESM1_ESM.pdf]

Supplementary Appendix

to

Expansion of Germline Variants in Primary Hyperparathyroidism: Krebs Cycle Defects as a Cause of Parathyroid Adenomas

Endocrine Pathology

Hussam Alkaissi[1], Elias Chuki[2], Yi Liu[3], James Welch[2], Lynn Bliss[2], Niharika Shah[4], Sunita K. Agarwal[2], William F. Simonds[2], Martha Quezado[4], Sanaz Sakiani[1], Thorkell Andresson[5], Karel Pacak[6], Naris Nilubol[7], Lee S. Weinstein[2], Christopher A. Febres-Aldana[4], Smita Jha[2]

[1]National Institute of Diabetes and Digestive and Kidney Diseases, National Institutes of Health, Bethesda, Maryland, USA 20892

[2]Metabolic Diseases Branch, National Institute of Diabetes and Digestive and Kidney Diseases, National Institutes of Health, Bethesda, Maryland, USA 20892

[3]Genetics Branch, Center for Cancer Research, National Cancer Institute, National Institutes of Health, Bethesda, MD, USA 20892

[4]Laboratory of Pathology, Center for Cancer Research, National Cancer Institute, Bethesda, Maryland, USA 20892

[5]Frederick National Laboratory for Cancer Research, National Institutes of Health, Frederick, Maryland, USA 21701

[6]Eunice Kennedy Shriver National Institute of Child Health and Human Development, Bethesda, Maryland, USA 20892

[7]Endocrine Surgery Section, Surgical Oncology Program, National Cancer Institute, Bethesda, Maryland, USA 20892

**Supplementary Table 1: Metabolites measured in the study**	
**Metabolite name**	**Abb**
Pyruvate	PYR
Lactate	LAC
Acetyl-coenzyme A	AcCoA
Citrate	CIT
Isocitrate	ISOCIT
Cis-aconitate	ACT
2-oxoglutarate	AKG
Succinate	SUC
Fumarate	FUM
Malate	MAL
Glutamine	GLN
Glutamic acid	Glu
Glucose-6-phosphate	G6P
Fructose-6-phosphate	F6P
Fructose-1,6-diphosphate	FBP
Dihydroxy-acetone-phosphate	DHAP
Glyceraldehyde-3-phosphate	GAP
3-phosphoglycerate	3PG
Phosphoenolpyruvate	PEP
6-phosphogluconate	6PG
Ribose-5-phosphate	R5P
Xylulose-5-phosphate	XYLU5P
Sedoheptulose-7-phosphate	S7P
Adenosine monophosphate	AMP
Adenosine diphosphate	ADP
Adenosine triphosphate	ATP
Guanosine triphosphate	GTP
Nicotinamide adenine dinucleotide	NAD
Nicotinamide adenine dinucleotide (reduced)	NADH
Nicotinamide adenine dinucleotide phosphate	NADP
Nicotinamide adenine dinucleotide phosphate (reduced)	NADPH
Flavin adenine dinucleotide	FAD
Glutathione	GSH

Supplementary Table 2: Concentration (µM) of metabolites normalized to protein (Batch 1)						
**Metabolite**	**MEN1-PTH1**	**MEN1-PTH2**	**MEN1-PTH3**	**DK-2231-1**	**FHd-PCC1**	**FHd-PCC2**
**PYR**	6.78	61.19	34.2	208	5.04	0.8
**LAC**	817.49	1985.49	3709.48	17445	696.52	381.38
**AcCoA**	0.06	0.42	0.76	2	0.07	0.06
**CIT**	47.5	37.26	92.71	203	12.3	6.19
**ACT**	12.13	4.49	24.57	51	2.73	1.6
**AKG**	0.3	3.31	5.24	27	2.22	0.88
**SUC**	7.62	30.76	108.05	803	37.79	21.19
**FUM**	25.64	56.76	66.87	4311	104.5	59.76
**MAL**	53.4	126.3	118.76	982	47.32	24.88
**2HG**	1.71	3	2.36	3	0.13	0.05
**GLN**	79.11	30.55	51.85	106	7.99	3.9
**GLU**	99.71	175.1	454.28	972	75.52	41.12
**G6P**	44.79	9.12	15.58	68	2.32	1.43
**F6P**	3.69	7.61	3.35	33	0.5	0.16
**FBP**	6.88	4.76	9.57	228	2.03	0.65
**DHAP**	1.24	2.43	3.67	8	0.14	0.06
**GAP**	14.2	7.68	22	42	1.4	0.9
**3PG**	118.94	71.88	145.85	440	7.06	3.83
**PEP**	15.69	10.91	19.73	110	1.21	0.42
**6PG**	1.82	4.12	6.68	84	0.52	0.52
**R5P**	3.79	2.94	2	7	0.31	0.05
**XYLU5P**	4.42	11.68	18.27	38	0.85	0.5
**S7P**	0.54	4.95	2.82	23	1.36	0.58
**AMP**	113.18	143.67	206.68	701	72.65	45.15
**ADP**	61.78	65.45	120.85	367	56.42	30.51
**ATP**	32.47	26.05	44.3	290	45.33	22.15
**GTP**	4.61	2.31	3.39	33	5.76	1.7
**NAD**	48.15	67.7	186.17	523	23.6	11.98
**NADH**	2.72	0.94	3.3	5	0.07	0.04
**NADP**	5.89	6.03	11.13	62	1.74	1.16
**NADPH**	1.03	0.11	0.19	1	0.01	0.01
**FAD**	2.02	2.34	6.22	6	0.29	0.26
**GSH**	4.96	182.83	408.59	772	64.73	34.57
**FMR**	0.48	0.45	0.56	4.39	2.21	2.4

Supplementary Table 3: Concentration (µM) of metabolites normalized to protein (Batch 2)						
**Metabolite**	**MEN1-PTH4**	**MEN1-PTH5**	**MEN1-PTH6**	**DK-2231-2**	**DK-2005**	**FHd-PCC3**
**PYR**	1.04	3.38	6.58	2.87	0.46	10.32
**LAC**	40.84	89.58	1.48	150.13	36.71	431.92
**AcCoA**	0.01	0.01	0	0.01	0.01	0.03
**CIT**	1.43	0.95	0.3	1.4	1.61	11.1
**ACT**	0	0	0	0	0	2.93
**AKG**	0.05	0.14	0.74	0.2	0.11	1.41
**SUC**	0.74	1.84	0.2	5.41	1.13	23.44
**FUM**	1.17	1.6	0.61	27.2	1.62	83
**MAL**	3.74	4.8	0.43	7.67	5.21	28.24
**2HG**	0.08	0.14	0.08	0.07	0.08	0.14
**GLN**	0.87	1.42	8.72	1.68	1.08	6.65
**GLU**	4.09	8.18	1.22	5.14	8.2	45.1
**G6P**	0.3	0.52	0.03	0.73	0.34	0.86
**F6P**	0.13	0.23	0.01	0.21	0.14	0.3
**FBP**	0.13	0.35	0.03	0.46	0.33	0.61
**DHAP**	0.16	0.43	0.02	0.34	0.13	0.05
**GAP**	0.15	0.17	0.03	0.04	0.17	0.75
**3PG**	0.57	0.93	0.15	0.94	0.55	2.02
**PEP**	0.05	0.06	0.06	0.07	0.04	0.86
**6PG**	0.12	0.25	0.07	0.35	0.16	0.3
**R5P**	0.29	0.77	0.04	0.19	0.44	0.48
**XYLU5P**	0.24	0.69	0.03	0.21	0.14	0.11
**S7P**	0.34	0.4	0.02	1.02	0.42	1.73
**AMP**	4.24	4.39	0.15	4.8	3.47	45.61
**ADP**	1.05	2.04	0.19	1.65	0.88	33.14
**ATP**	0.37	1.68	0.12	0.83	0.34	31.21
**GTP**	0.02	0.07	0.01	0.04	0.03	2.92
**NAD**	1.69	2.2	0.08	2.52	1.6	11.44
**NADH**	0.03	0.02	0.01	0.03	0.02	0.06
**NADP**	0.09	0.11	0.02	0.15	0.09	0.64
**NADPH**	0.01	0.01	0.01	0.01	0.01	0.01
**FAD**	0.05	0.07	0.01	0.07	0.05	0.29
**GSH**	12.62	13.99	0.11	15.73	14.53	47.13
**FMR**	0.31	0.33	1.42	3.55	0.31	2.94

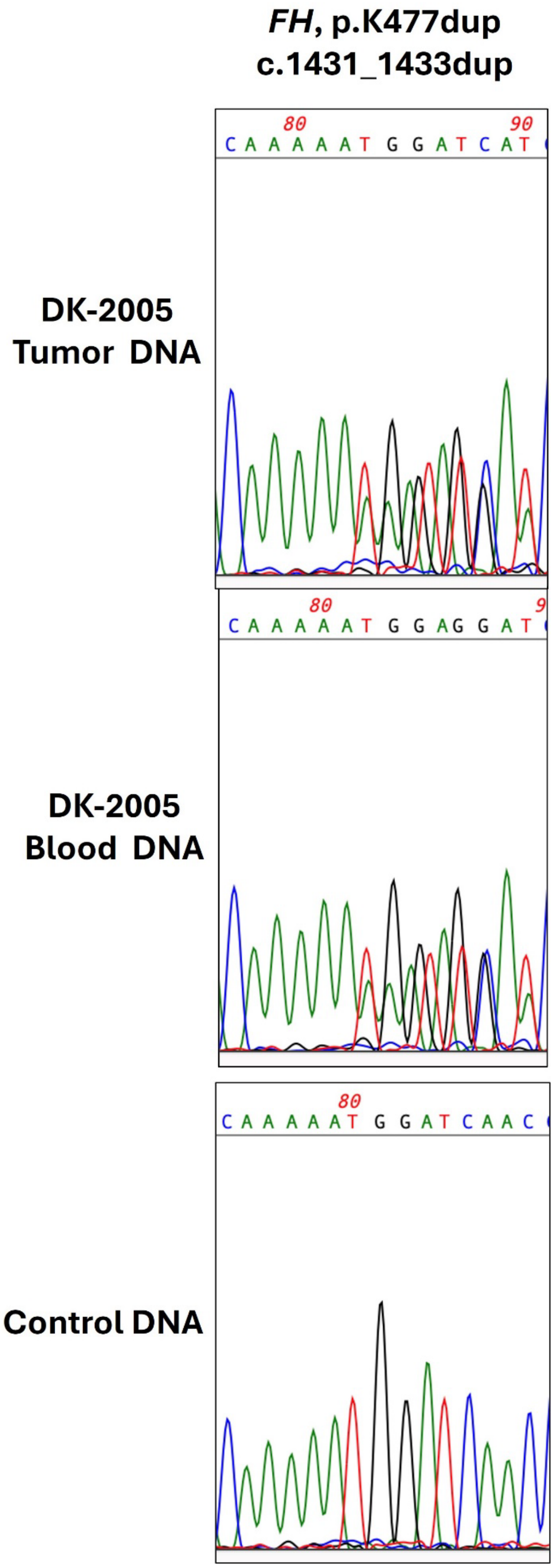

Supplementary Figure 1: Chromatograms showing sequence of *FH* variant p.K477dup in parathyroid tumor DNA and blood DNA of patient DK-2005, compared to a control DNA sample. Loss of heterozygosity is not observed in the tumor DNA due to the lack of predominance of variant allele peaks compared to blood DNA.
